# Supplementary material for: Nc886, a Novel Suppressor of the Type I Interferon Response Upon Pathogen Intrusion
Source: Int J Mol Sci. 2021 Feb 18;22(4):2003. doi: 10.3390/ijms22042003 (PMC7922379; doi:10.3390/ijms22042003)
Supplement: Supplementary file 1 [file ijms-22-02003-s001.pdf]

Supplementary Information (Figure S1-S2)

(Lee, et al: "nc886, a novel suppressor of the type I interferon response upon pathogen intrusion")

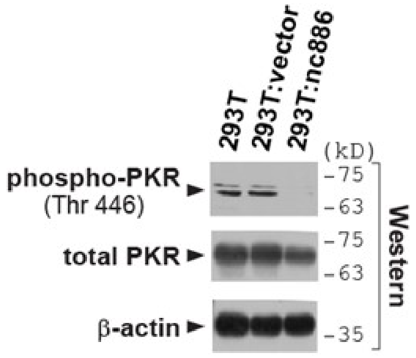

Figure S1. The basal level of PKR activity is lower in nc886-expressing cells

Western blot of indicated proteins. Molecular size markers are on the right. The exponentially growing cells without any stimulus were harvested for Western. The basal level of PKR activation, as seen by phospho-PKR, was lower in nc886-expressing cells ("293T:nc886") than the original 293T cells or control cells ("293T:vector") lacking nc886 expression

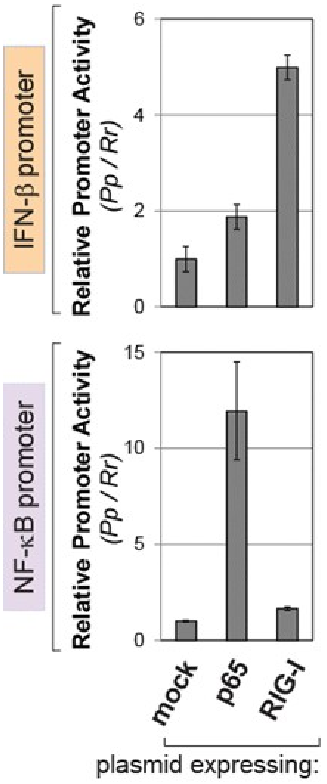

Figure S2. Ectopic expression of RIG-I induces the IFN-β promoter, whereas that of NF-κB p65 does not.

Measurement of the activity of luciferase whose expression is driven by promoters indicated on the left. Plasmids expressing NF-κB p65 (Koh *et al.*, *Mol. Cells* 2009, 28, 553-558) or RIG-I (Ren *et al.*, *J. Virol.* 2012, 86, 13049-13061) were co-transfected with luciferase-expressing plasmids, followed by cell harvest at 24 hr post-transfection. Transfection of plasmids, luciferase assays, and plotting of data were as described in the main text (see "Materials and Methods" and Fig 1D legend).

Ectopic expression of RIG-I was sufficient to induce the IFN-β promoter. In comparison, the effect of NF-κB p65 was very marginal (< 2-fold; "p65" in upper panel). In a control experiment employing a NF-κB-responsive promoter, NF-κB p65 induced the promoter robustly, ensuring the expression of NF-κB p65, but RIG-I barely affected. In conclusion, the expression of RIG-I (which leads to IRF3 activation; see Fig 2A), but not that of NF-κB p65 was sufficient to activate the IFN-β promoter. This result corroborated previous notion that IRF-3 plays a pivotal role, while NF-κB and AP-1 are necessary for the IFN-β promoter to be fully activated (Kim and Maniatis, *Mol. Cell* 1997, 1, 119-129).
